# Niacin supplementation increases the number of oxidative type I fibers in skeletal muscle of growing pigs

**DOI:** 10.1186/1746-6148-9-177

**Published:** 2013-09-09

**Authors:** Muckta Khan, Robert Ringseis, Frank-Christoph Mooren, Karsten Krüger, Erika Most, Klaus Eder

**Affiliations:** 1 Institute of Animal Nutrition and Nutrition Physiology, Justus-Liebig-University Giessen, Heinrich-Buff-Ring 26-32, Giessen 35390, Germany; 2 Department of Sports Medicine, Justus-Liebig-University Giessen, Kugelberg 62, Giessen 35394, Germany

**Keywords:** Niacin, Pig, Muscle fiber transition, Oxidative type I fiber

## Abstract

**Background:**

A recent study showed that niacin supplementation counteracts the obesity-induced muscle fiber switching from oxidative type I to glycolytic type II and increases the number of type I fibers in skeletal muscle of obese Zucker rats. These effects were likely mediated by the induction of key regulators of fiber transition, PGC-1α and PGC-1β, leading to muscle fiber switching and up-regulation of genes involved in mitochondrial fatty acid import and oxidation, citrate cycle, oxidative phosphorylation, mitochondrial biogenesis. The aim of the present study was to investigate whether niacin supplementation causes type II to type I muscle and changes the metabolic phenotype of skeletal muscles in growing pigs.

**Results:**

25 male, 11 wk old crossbred pigs (Danzucht x Pietrain) with an average body weight of 32.8 ± 1.3 (mean ± SD) kg were randomly allocated to two groups of 12 (control group) and 13 pigs (niacin group) which were fed either a control diet or a diet supplemented with 750 mg niacin/kg diet. After 3 wk, the percentage number of type I fibers in three different muscles (*M. longissismus dorsi*, *M. quadriceps femoris*, *M. gastrocnemius*) was greater in the niacin group and the percentage number of type II fibers was lower in the niacin group than in the control group (*P* < 0.05). The mRNA levels of PGC-1β and genes involved in mitochondrial fatty acid catabolism (CACT, FATP1, OCTN2), citrate cycle (SDHA), oxidative phosphorylation (COX4/1, COX6A1), and thermogenesis (UCP3) in *M. longissimus dorsi* were greater in the niacin group than in the control group (*P* < 0.05).

**Conclusions:**

The study demonstrates that niacin supplementation induces type II to type I muscle fiber switching, and thereby an oxidative metabolic phenotype of skeletal muscle in pigs. Given that oxidative muscle types tend to develop dark, firm and dry pork in response to intense physical activity and/or high psychological stress levels preslaughter, a niacin-induced change in the muscle´s fiber type distribution may influence meat quality of pigs.

## Background

Niacin, also called nicotinic acid, is a water-soluble vitamin which belongs to the vitamin B complex and is essential for the metabolism of carbohydrates, fats and many other substances. At pharmacological doses, niacin exerts pronounced lipid-lowering activities, particularly on triacylglycerols (TAG), but also on total cholesterol and LDL cholesterol [1], and, interestingly, niacin increases HDL cholesterol [2]. Besides these well-documented effects on blood lipid profile, high doses of niacin were also shown to cause profound, but less recognized changes in gene expression in several tissues [3]. In this regard it is worth mentioning, that studies in both, humans [4] and rats [5] revealed that high levels of niacin cause an up-regulation of transcription factors in skeletal muscles, like PPARγ coactivator-1α (PGC-1α) and PGC-1β, which are key regulators of fiber distribution in skeletal muscle [6,7]. In principle, two major fiber types of skeletal muscle can be distinguished: type II fibers, also called glycolytic fibers, which have few mitochondria and largely generate ATP through glycolytic metabolism, and type I fibers, also called oxidative fibers, which are mitochondria-rich and utilize mainly oxidative phosphorylation for energy production [8,9]. Remarkably, the type II to type I fiber distribution in a given muscles displays high plasticity and can be induced to switch depending on various factors, like exercise, mechanical unloading or obesity [10-13]. Since the muscle´s fiber distribution determines its metabolic phenotype, fiber switching induced by exercise, mechanical unloading or obesity results in a change of the functional and metabolic phenotype of skeletal muscle [10-13]. Based on the observation that niacin up-regulates key regulators of fiber switching in skeletal muscle, it has been investigated whether niacin supplementation can prevent the obesity-induced muscle fiber switching from type I to type II and causes an elevation in the number of type I fibers in skeletal muscle of obese Zucker rats [5]. This study indeed showed that niacin prevents the obesity-induced muscle fiber switching from type I to type II and elevates the number of type I fibers in skeletal muscle of obese Zucker rats [5]. Corresponding to this niacin-induced increase in the muscle´s type I fiber content niacin supplementation to the obese Zucker rats caused the development of a more oxidative metabolic phenotype of skeletal muscle as evidenced by an increased expression of genes involved in mitochondrial fatty acid import and oxidation, citrate cycle, oxidative phosphorylation, mitochondrial biogenesis and angiogenesis [5]. This obvious improvement in the muscle´s capacity for oxidative utilization of fatty acids has likely contributed, at least partially, to the strong lowering effect of niacin on blood levels of TAG and non-esterified fatty acids (NEFA) in the obese Zucker rats [5], which are characterized by markedly elevated blood levels of TAG and NEFA.

It is currently unknown whether high levels of niacin also causes type II to type I muscle fiber switching in metabolically healthy animals. This question may be of particular interest in farm animals used for meat production like growing pigs because a change in the muscle´s fiber type distribution is expected to influence meat quality considering that several studies have reported that oxidative muscles with a high percentage of type I fibers have a lower glycolytic potential [14,15], a darker color [16,17] and a higher ultimate pH [14,16]. In addition, it was shown that oxidative muscle types tend to develop dark, firm and dry pork in response to intense physical activity and/or high psychological stress levels preslaughter [18]. Therefore, the present study aimed to investigate whether niacin supplementation causes type II to type I muscle fiber switching, thereby, resulting in an increased type I fiber percentage in skeletal muscle of growing pigs.

## Methods

### Animals, housing, and experimental design

The experiment was performed at the Institute of Animal Nutrition and Nutrition Physiology, University of Giessen, Germany. A total of 25 male, 11 wk old crossbred pigs (Danzucht × Pietrain) with an average body weight of 32.8 ± 1.3 (mean ± SD) kg were randomly allocated to two groups of 12 (control group) and 13 pigs (niacin group), respectively. The pigs were kept individually in pens in a room under controlled temperature at 23 ± 2°C and relative humidity at 55 to 60% with light from 06.00 to 18.00 hrs. Both groups of pigs received a nutritionally adequate commercial diet (RWZ-UNIVERSAL-START HE Press, RWZ, Köln, Germany) for growing pigs containing (in g/kg) wheat (226), barley (200), soybean meal (149), triticale (100), corn (100), wheat gluten (100), dried distiller´s grains (31), rapeseed meal (20), wheat bran (20), calcium carbonate (16.1), vegetable oil (10), sodium chloride (3.9), monocalcium phosphate (2), and vitamin-mineral premix (22). The vitamin-mineral premix provided 34 mg of niacin per kg diet, a dose which is sufficient to meet the niacin requirement of growing pigs [19]. In the niacin group, the commercial diet was supplemented with additional 750 mg of niacin (obtained from Lonza, Basel, Switzerland) per kg as a pharmacological dose. The diets and water were given ad libitum. The feeding experiment lasted 21 days. All experimental procedures were in strict accordance with the recommendations in the guidelines for the care and use of laboratory animals [20] and the Appendix A of European Convention for the Protection of Vertebrate Animals used for Experimental and other Scientific Purposes. In accordance with article 4 par. 3 of the German Animal Welfare Law all animals were humanely killed for scientific purpose approved by the Animal Welfare Officer of the Justus-Liebig-University.

### Sample collection

After 21 days the animals were slaughtered after a 12 h fasting period at a commercial slaughterhouse near by the Institute. Blood samples were taken into EDTA polyethylene tubes (Sarstedt, Nürnbrecht, Germany) and plasma was collected by centrifugation (1,100 × g; 10 min, 4°C). Samples from three different skeletal muscles [*M. longissimus dorsi* (LD), *M. quadriceps femoris* (QF), *M. gastrocnemius* (G)] were excised and samples were shock frozen with liquid nitrogen and stored at −80°C pending analysis.

### Determination of type I and type II fiber percentages in skeletal muscle

Determination of type I and type II fiber percentages in skeletal muscle was carried out as recently described in detail [5]. In brief, 30 μm thick, serial cross sections were prepared using a cryostat microtome, mounted on cover slips and stained for myosin ATPase (mATPase) using a modified method of Hämäläinen and Pette [21]. Subsequently, the sections were analyzed by light microscopy (Leica DMI 6000B) for calculating the type I and type II fiber percentages.

### Determination of TAG and NEFA concentrations in plasma

Concentrations of TAG and NEFA in plasma were determined by enzymatic reagent kits from Merck Eurolab (ref. 113009990314) and from Wako Chemicals (ref. RD291001200R), respectively.

### Determination of nicotinic acid and nicotineamide concentrations in plasma

Concentrations of nicotinic acid and nicotineamide in plasma were determined by LC-MS/MS according to the method from Liu et al. [22].

### RNA isolation, cDNA synthesis and qPCR analysis

RNA isolation, cDNA synthesis and qPCR analysis were performed as described recently in detail [23]. In brief, total RNA was extracted from 50–60 mg skeletal muscle aliquots using peqGOLD TriFast™ RNA Extraction reagent (Peqlab, Erlangen, Germany) according to the manufacturer´s protocol, and RNA concentration and purity were estimated from the optical density at 260 and 280 nm (Infinite 200 M microplate reader, Tecan, Männedorf, Switzerland). cDNA synthesis was carried out within one week after RNA isolation using dT18 primer and M-MuLV Reverse Transcriptase (MBI Fermentas, St. Leon-Rot, Germany). qPCR analysis was performed using KAPA SYBR FAST qPCR Universal Mastermix (Peqlab, Erlangen, Germany) and gene-specific primer pairs which are listed in Table 1. Calculation of gene expression data and normalization by GeNorm normalization factor were carried out as described recently [23]. The normalization factor was calculated as the geometric mean of expression data of the three most stable out of five tested potential reference genes. Means and SD were calculated from normalized expression data for samples of the same treatment group. The mean of the group control group was set to 1 and mean and SD of the niacin group were scaled proportionally. Data on qPCR performance for genes measured in skeletal muscle are shown in Table 1.

**Table 1:** Characteristics and performance data of primers used for qPCR

**Gene**	**Forward primer (3′-5′)**	**Product length (bp)**	**NCBI Genbank**	**Slope**	**R**^**2#**^	Efficiency*
	**Reverse primer (5′-3′)**					
*Reference genes*						
ATP5G1	CAGTCACCTTGAGCCGGGCGA	94	NM_001025218	−3.42	0.999	1.96
	TAGCGCCCCGGTGGTTTGC					
ACTB	GACATCCGCAAGGACCTCTA	205	XM_003124280	−3.60	0.998	1.89
	ACATCTGCTGGAAGGTGGAC					
RPS9	GTCGCAAGACTTATGTGACC	325	XM_003356050	−3.64	0.999	1.88
	AGCTTAAAGACCTGGGTCTG					
*Target genes*						
COX4/1	GTGGAACTGTACCGCCTGAA	257	XM_003355730	−3.44	1.000	1.95
	TTGTCGTAGTCCCACTTGGC					
COX6A1	CTCAGCTCGCATGTGGAAGA	139	NM_001190221	−3.34	0.996	1.99
	GATGCGAAGATGGGGGTAGG					
CACT/SLC25A20	GCAAAGCCCATTAGCCCTCT	235	XM_003483178	−3.21	0.988	2.05
	GAGCACATCCTCTGGGTGTT					
PPARGC1A	TAAAGATGCCGCCTCTGACT	168	NM_213963	−3.94	0.993	1.79
	TGACCGAAGTGCTTGTTCAG					
PPARGC1B	AAGTGCGGCTTCGTCACCTA	216	XM_003124093	−3.28	0.998	2.02
	GCTGTCGAAATCCATGGCTT					
SLC22A5	TGCATTTGGCTACATGCTGC	174	XM_003123912	−3.76	0.995	1.85
	ATGATCACCTCAGCTTCCTG					
SDHA	CTACGCCCCCGTCGCAAAGG	380	DQ402993	−3.24	1.000	2.03
	AGTTTGCCCCCAGGCGGTTG					
MYH2	GGCCCTTTGATGCCAAGACA	188	NM_214136	−3.45	1.000	1.95
	GGCCATGTCCTCGATCTTGT					
MYH4	GTGCCCTGCTGCCATCAATA	363	NM_001123141	−3.53	1.000	1.92
	TGCGTAACGCTCTTTGAGGT					
MYH7	TGCCAGCTTGAGCCTCTTTC	380	NM_213855	−3.33	0.999	2.00
	GTAGCGCTCCTTGAGGTTGT					
FATP1	GGTTCCAGCCTGTTGAATGT	275	NM_001083931	−3.44	0.990	1.95
	AACAAAACCTTGGTGCTTGG					
UCP2	AGTGTGAGACCTGACGAAGC	435	NM_214289	−3.64	0.996	1.88
	GCTTGACGGAGTCGTAGAGG					
UCP3	GCCACTTTGTCTCTGCCTTC	219	NM_214049	−3.49	0.998	1.93
	CAAACATCACCACGTTCCAG					

^#^Coefficient of determination of the standard curve.

*The efficiency is determined by [10^(−1/-slope^].

### Statistical analysis

Data were statistically analysed by one-way ANOVA using the Minitab Statistical Software (Rel. 13.0, State College, PA, USA). Means of the two groups were compared by Fisher’s multiple range test. Means were considered significantly different for *P* < 0.05. Data presented are shown as means ± SD.

## Results

### Feed intake, body weight development, carcass weights and feed conversion ratios

Feed intake, initial and final body weights, total and daily body weight gain, carcass weights and feed conversion ratio did not differ between the control group and the niacin group (Table 2).

**Table 2:** Feed intake, body weight gain, feed conversion ratio and carcass weight of pigs fed either a control diet or a diet supplemented with 750 mg niacin/kg diet for 3 wk

	**Control**	**Niacin**	***P *value**
	**n = 12**	**n = 13**	**(ANOVA)**
Feed intake (kg/d)	2.14 ± 0.27	2.13 ± 0.26	0.838
Initial body weight (kg)	32.7 ± 1.3	32.9 ± 1.5	0.829
Final body weight (kg)	53.5 ± 2.4	53.7 ± 3.9	0.864
Total body weight gain (kg)	20.7 ± 2.2	20.9 ± 2.6	0.915
Daily body weight gain (kg)	0.99 ± 0.10	0.99 ± 0.13	0.915
Carcass weight (kg)	40.5 ± 1.9	41.0 ± 2.9	0.567
Feed conversion ratio (kg feed/kg weight gain)	2.21 ± 0.42	2.22 ± 0.42	0.899

Values are means ± SD.

### Concentrations of nicotinic acid and its metabolite nicotineamide in plasma

The plasma concentrations of nicotinic acid (NA) and its metabolite nicotineamide (NAM) were greater in the niacin group than in the control group (NA: < 5 ng/mL (limit of detection) vs. 32.0 ± 13.0 ng/mL; NAM: 0.34 ± 0.07 vs. 3.88 ± 2.02 μg/mL; control group vs. niacin group; *P* < 0.05).

### Concentrations of TAG and NEFA in plasma

In order to assess whether lipid concentrations in plasma are influenced by niacin supplementation, we measured the concentrations of TAG and NEFA in plasma of the pigs. The plasma concentrations of both, TAG and NEFA were not different between the two groups of pigs (TAG: 0.51 ± 0.12 vs. 0.51 ± 0.10 mmol/L; NEFA: 0.47 ± 0.27 vs. 0.65 ± 0.21 mmol/L; control group vs. niacin group).

### Fiber type distribution of different skeletal muscles

**Figure 1:**
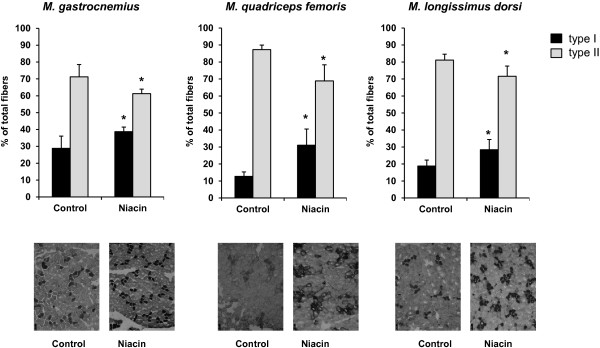
**Muscle fiber type distribution of *M. gastrocnemius*, *M. quadriceps femoris *and *M. longissimus dorsi *of pigs fed either a control diet or a diet supplemented with 750 mg niacin/kg diet for 3 wk.** Bars represent means ± SD, n = 12 (control) and 13 (niacin) pigs/group. Images from cross sections representing one animal per group are shown at the bottom. Asterisk denotes difference from control group, *P* < 0.05.

To study whether niacin supplementation causes type II to type I fiber switching, we determined the fiber type distribution in different skeletal muscles (LD, QF and G). The percentage number of type I fibers in all three muscles considered was greater in the niacin group than in the control group, whereas the percentage number of type II fibers was less in niacin group than in the control group (*P* < 0.05, Figure 1).

### Transcript levels of genes encoding fiber-specific MHC isoforms and regulators of muscle fiber distribution in LD muscle

In order to explore whether the niacin-induced fiber switching is reflected by changes in the expression of fiber-specific MHC isoforms, we determined the transcript levels of different MHC isoforms, from which three isoforms exist in pigs, namely one type I isoform (MHCI encoded by MYH7) and two type II isoforms (MHCIIA encoded by MYH2, and MHCIIB encoded by MYH4), in LD muscle. In line with the decreased type II fiber percentage the transcript levels of MYH2 and MYH4, which are expressed in type II fibers, were significantly reduced (*P* < 0.05) or tended to be reduced (*P* < 0.15), respectively, in LD muscle in the niacin group compared to the control group (Table 3). The transcript level of the MHC isoform MYH7, which is expressed in type I fibers, in LD muscle tended to be increased in the niacin group compared to the control group (*P* < 0.15, Table 3).

**Table 3:** Transcript levels of genes encoding fiber-specific MHC isoforms and regulators of muscle fiber distribution in LD muscle of pigs fed either a control diet or a diet supplemented with 750 mg niacin/kg diet for 3 wk

	**Control**	**Niacin**	***P *value**
	**n = 12**	**n = 13**	**(ANOVA)**
	**Relative mRNA level (fold of control)**		
*Fiber-specific MHC isoforms*			
MYH7 (type I-specific)	1.00 ± 0.29	1.26 ± 0.42	0.139
MYH2 (type IIA-specific)	1.00 ± 0.40	0.65 ± 0.21	0.023
MYH4 (type IIB-specific)	1.00 ± 0.51	0.67 ± 0.27	0.086
*Regulators of fiber distribution*			
PGC-1α	1.00 ± 0.91	1.49 ± 0.98	0.309
PGC-1β	1.00 ± 0.24	1.33 ± 0.31	0.021

Values are means ± SD.

To elucidate the mechanisms underlying type II to type I fiber transition in skeletal muscle of pigs in response to niacin supplementation, we determined the transcript levels of two key regulators of muscle fiber transition, PGC-1α and PGC-1β, in LD muscle. The transcript level of PGC-1β in LD muscle was greater in the niacin group than in the control group (*P* < 0.05; Table 3). In addition, the transcript level of PGC-1α in LD muscle was numerically greater in the niacin group than in the control group but this effect was not significant (*P* > 0.05; Table 3).

### Transcript levels of genes involved in fatty acid utilization, citrate cycle, oxidative phosphorylation and thermogenesis in LD muscle

Given that induction of PGC-1α and PGC-1β results in the induction of genes involved in mitochondrial fatty acid catabolism (CACT, FATP1, OCTN2), citrate cycle (SDHA), oxidative phosphorylation (COX4/1, COX6A1), and thermogenesis (UCP2, UCP3), we determined transcript levels of genes representing these pathways in LD muscle. The transcript levels of CACT, FATP1, OCTN2, SDHA, COX4/1, COX6A1 and UCP3 in LD muscle were greater in the niacin group than in the control group (*P* < 0.05; Table 4). The transcript level of UCP2 in LD muscle tended to be elevated in the niacin group compared to the control group (*P* < 0.15; Table 4).

**Table 4:** Transcript levels of genes involved in fatty acid utilization, citrate cycle, oxidative phosphorylation and thermogenesis in LD muscle of pigs fed either a control diet or a diet supplemented with 750 mg niacin/kg diet for 3 wk

	**Control**	**Niacin**	***P *value**
	**n = 12**	**n = 13**	**(ANOVA)**
	**Relative mRNA level (fold of control)**		
*Fatty acid utilization*			
CACT	1.00 ± 0.43	1.55 ± 0.62	0.038
FATP1	1.00 ± 0.31	1.33 ± 0.27	0.020
OCTN2	1.00 ± 0.31	1.44 ± 0.44	0.018
*Citrate cycle*			
SDHA	1.00 ± 0.34	1.53 ± 0.58	0.037
*Oxidative phosphorylation*			
COX4/1	1.00 ± 0.28	1.40 ± 0.45	0.033
COX6A1	1.00 ± 0.36	1.57 ± 0.62	0.022
*Thermogenesis*			
UCP2	1.00 ± 0.63	1.53 ± 0.84	0.130
UCP3	1.00 ± 0.45	1.46 ± 0.49	0.036

Values are means ± SD.

## Discussion

The main finding of the present study is that supplementation of a pharmacological niacin dose, similar with that recently used in Zucker rats (Pigs: 30–49 mg/kg body weight; Zucker rats: 40–54 mg/kg body weight [5]) in pigs causes type II to type I muscle fiber switching, thereby, resulting in an increased type I fiber percentage in skeletal muscle in comparison to pigs receiving a diet with a nutritionally adequate niacin concentration. In contrast to our study in Zucker rats, in which fiber distribution of only one muscle (M*. rectus femoris*) was studied, we analyzed fiber distribution of three different skeletal muscles (LD, QF and G) in the pigs in the present study. These muscles contained predominantly type II fibers but varied in their type II to type I fiber type ratios (control group: 4.6, 7.6 and 2.8 for LD, QF and G, respectively). We observed that niacin supplementation decreased this ratio in all three muscles considered (niacin group: 2.8, 2.6 and 1.6 for LD, QF and G, respectively) indicating that niacin exerts its effect on muscle fiber distribution independently of the muscle type, which extends our knowledge with regard to the effect of niacin supplementation on muscle fiber distribution. This effect was also reflected by a reduced expression of the type II fiber-specific transcript levels of MYH2 (*P* < 0.05) and MYH4 isoform (*P* < 0.15) and an increased expression of the type I fiber-specific isoform MYH7 (*P* < 0.15) in LD muscle of the niacin group compared to the control group.

Muscle fiber switching was reported to be initiated through the up-regulation of key regulators of muscle fiber distribution and muscle metabolic phenotype [6,24-26], and we have recently shown that niacin supplementation causes an up-regulation of two of these key regulators, namely PGC-1α and PGC-1β, in rectus femoris muscle of rats [5]. Like in rats, we observed in the present study that the transcript level of PGC-1β was elevated in LD muscle of pigs of the niacin group. In addition, the transcript level of PGC-1α in LD muscle was also increased in pigs of the niacin group, even though this effect was not significant, which is attributed to the relatively high standard deviation of this parameter in both groups of pigs. PGCs regulate the muscle metabolic phenotype by binding to and activating a variety of nuclear receptors and additional transcription factors. For example, PGC-1α dramatically co-activates PPARα and/or PPARδ in various cell types and tissues and thereby induces the expression of genes involved in fatty acid catabolism and thermogenesis. Similarly, co-activation by PGC-1α and PGC-1β has also been shown for the myocyte enhancer factor 2 family of transcription factors, which stimulate specifically the expression of MHC genes from oxidative fibers [25,27], and for nuclear respiratory factor-1 and estrogen-related receptor α, which are required for oxidative phosphorylation and mitochondrial biogenesis [28]. In line with the up-regulation of key regulators of type II to type I fiber switching in LD muscle, we observed that pigs of the niacin group had elevated transcript levels of genes involved in mitochondrial fatty acid catabolism (CACT, FATP1, OCTN2), citrate cycle (SDHA), oxidative phosphorylation (COX4/1, COX6A1), and thermogenesis (UCP3) in LD muscle. All these genes are abundantly expressed in type I fibers, which is responsible for the oxidative metabolic phenotype and the preferred utilization of oxidative phosphorylation for energy production of type I fibers [8,29]. Thus, the abovementioned changes in gene expression in LD muscle of pigs of the niacin group are consistent with the niacin-induced increase of type I fiber content in LD muscle. It is currently unknown how niacin mediates the observed up-regulation of key regulators of skeletal muscle phenotype because the skeletal muscle does not express the niacin receptor. This suggests that the effect of niacin involves niacin receptor-independent mechanisms. In this context it noteworthy that niacin has been reported recently to induce several humoral changes, like increases in the plasma levels of epinephrine, corticosterone and glucagon [30]. In addition, niacin supplementation also causes an elevation in the plasma levels of growth hormone, adiponectin and leptin [31,32], all of which are well-documented to influence gene expression and cellular signaling in different tissues. Thus, future studies have to clarify whether these niacin-induced humoral changes are responsible for the observed muscle fiber switching.

In contrast to our recent study in obese Zucker rats [5] niacin supplementation did not induce the well-documented plasma TAG-lowering effect in pigs. The lack of effect, however, is probably not due to an insufficient niacin dose because the dose was similar as in our rat study [5] and the administered niacin dose caused a significant increase in plasma nicotinic acid and particularly nicotineamide levels indicating sufficient bioavailability. It is more likely that plasma TAG concentration of pigs was not lowered because it was yet within the normal range making a further reduction unlikely. In addition, in opposite to the well-documented antilipolytic effect of niacin [1] the plasma NEFA concentration in pigs of the niacin group was also not reduced but even increased, at least numerically. This result, however, is in agreement with recent observations that chronic niacin administration for at least 2 weeks results in elevated plasma NEFA levels [33]. The basis for this rebound phenomenon on lipolysis during long-term niacin treatment is only incompletely understood, but recent findings indicate that niacin favors an increase in the net rate of lipolysis through reducing TAG synthesis and expression of perilipin in adipocytes [34].

## Conclusions

The present study demonstrates that niacin supplementation induces type II to type I muscle fiber switching, and thereby an oxidative metabolic phenotype of skeletal muscle in pigs as a farm animal model. The observed up-regulation of key regulators of fiber distribution in skeletal muscle in response to niacin supplementation is likely causative for the induction of muscle fiber switching in pigs. Given that oxidative muscle types tend to develop dark, firm and dry pork in response to intense physical activity and/or high psychological stress levels preslaughter [18], a niacin-induced change in the muscle´s fiber type distribution may influence meat quality of pigs which would be worth of being investigated in future studies.

## Abbreviations

CACT: Carnitine-acylcarnitine translocase; COX: Cytochrome c oxidase; FATP: Fatty acid transport protein; G: Gastrocnemius; HDL: High-density lipoprotein; LD: Longissimus dorsi; LDL: Low-density lipoprotein; MHC: Myosin heavy chain; MYH: Myosin heavy chain encoded gene; NA: Nicotinic acid; NAM: Nicotineamide; NEFA: Non-esterified fatty acids; OCTN: Novel organic cation transporter; PGC-1: Peroxisome proliferator-activated receptor-gamma coactivator-1; PPAR: Peroxisome proliferator-activated receptor; QF: Quadriceps femoris; SDHA: Succinate dehydrogenase subunit A; TAG: Triacylglycerol; UCP: Uncoupling protein.

## Competing interests

The authors declare that they have no competing interests.

## Authors’ contributions

MK conducted the animal experiment, performed fiber typing, PCR analyses, blood lipid analyses, statistical analyses, and wrote the manuscript, RR participated in the design and coordination of the study, supervised PCR analyses, and statistical analysis and helped to draft the manuscript, FCM and KK analysed data from muscle fiber typing, EM performed nicotinic acid and nicotineamide analyses in blood, KE conceived of the study, participated in its design and coordination and helped to draft the manuscript. All authors read and approved the final manuscript.
